# Research progress in deep learning-based fundus image analysis for the diagnosis and prediction of hypertension-related diseases

**DOI:** 10.3389/fcell.2025.1608994

**Published:** 2025-08-06

**Authors:** Ping Li, Juan Tao, Quan Yuan, Rongqing Zhang, Peng Gao

**Affiliations:** ^1^ Department of Ophthalmology, Shanghai Tenth People’s Hospital of Tongji University, Tongji University School of Medicine, Shanghai, China; ^2^ School of Computer Science and Technology, Tongji University, Shanghai, China

**Keywords:** hypertension-related diseases, fundus imaging, retinal vasculature, color fundus photography, artificial intelligence, deep learning

## Abstract

Hypertension-related diseases have widespread effects on the systemic microvasculature, with particularly significant impacts on the retinal vascular system. As a non-invasive window to observe vascular abnormalities, fundus imaging plays an important role in the diagnosis and prediction of hypertension-related conditions. In recent years, deep learning (DL) has rapidly advanced in the field of color fundus photography (CFP) analysis, demonstrating strong potential in vessel segmentation, artery/vein classification, lesion detection, and systemic disease prediction. This review systematically summarizes recent progress in DL-based fundus analysis for hypertension-related diseases, focusing on hypertensive retinopathy analysis, automated diagnosis of ocular conditions, and cardiovascular risk prediction. Studies have shown that DL can accurately extract retinal vascular structures and pathological features, offering reliable support for early screening and risk stratification of hypertension-related diseases. Nonetheless, current models still face challenges in generalizability, robustness to low-quality images, and clinical interpretability. Future research should emphasize multimodal data integration, lightweight model design, and clinical validation to promote the real-world application of these technologies in the management of hypertension-related diseases.

## 1 Introduction

Hypertension is a prevalent systemic condition with profound impacts on multiple organs, particularly the microvascular system. Chronic hypertension causes vascular wall thickening and sclerosis, resulting in reduced blood supply and damage to vital organs, such as the heart, brain, and kidneys. In the retina, hypertension induces characteristic changes, including variations in vascular caliber, arteriovenous nicking, retinal hemorrhages, and optic disc edema, which may progress to hypertensive retinopathy (HR) (Cheung et al., 2012; Wong and Mitchell, 2004). Retinal changes are influenced by factors such as patient age, blood pressure elevation severity, and hypertension duration. Conditions like resistant hypertension, hypertensive emergencies, and secondary hypertension, such as hypertensive disorders of pregnancy (HDP), are often accompanied by retinal abnormalities. Additionally, hypertension is a major risk factor for retinal diseases, including retinal vein occlusion and retinal artery occlusion (Wong and Mitchell, 2007).

Fundus imaging is a fundamental diagnostic tool in ophthalmology, color fundus photography (CFP) is the most commonly used imaging modality in ophthalmology. It captures high-resolution, two-dimensional color images of the retina and serves as the primary data source in the majority of ophthalmic studies. This technique captures a two-dimensional projection of the retina using a monocular camera, producing high-resolution images. Compared to other imaging modalities like optical coherence tomography (OCT) and fundus fluorescein angiography (FFA), fundus imaging is non-invasive, convenient, and cost-effective, making it suitable for large-scale screening (Bruce et al., 2011; Rodenbeck and Mackay, 2019; Romero-Oraa et al., 2020). By identifying retinal changes such as vascular narrowing, arteriovenous nicking, retinal hemorrhages, and optic disc edema, fundus imaging enables early diagnosis and timely intervention at each stage of disease to prevent disease progression, making it invaluable for managing these conditions (Fraser-Bell et al., 2017).

Recent advancements in artificial intelligence (AI), particularly deep learning (DL) algorithms, have introduced powerful tools for automating CFP analysis and enhancing diagnostic efficiency. By simulating human neural networks, DL processes complex medical image data, identifies specific pathological features, and significantly improves diagnostic accuracy (LeCun et al., 2015). DL has been applied across various medical disciplines, especially in image-dependent fields such as pathology, radiology, ophthalmology, and dermatology (Ting et al., 2019a; Jiang et al., 2020). These algorithms enable rapid and precise disease diagnosis by analyzing large-scale datasets, automating feature extraction, and interpreting key biomarkers. For example, DL models achieve diagnostic accuracy comparable to human experts in detecting tuberculosis from chest X-rays (Lakhani and Sundaram, 2017; Ting et al., 2018) and distinguishing malignant melanoma from benign lesions in dermatology (Esteva et al., 2017).

In ophthalmology, DL algorithms have shown remarkable success, particularly in diagnosing diseases using CFPs. By training on extensive datasets, these algorithms learn to identify disease-specific patterns and features, enabling rapid diagnosis and grading. AI-driven DL techniques reduce the workload of ophthalmologists by automating preliminary screenings and classifications. Unlike human judgment, which may be influenced by experience and bias, DL relies on objective data, ensuring consistency and reliability in diagnostics (Ting et al., 2019b; Li et al., 2021). Applications of DL in ophthalmology include early screening and diagnosis of diabetic retinopathy, glaucoma, and macular degeneration. For instance, DL models can detect and classify microaneurysms, hemorrhages, and exudates in CFP, assessing disease severity to guide treatment strategies (Ting et al., 2017; Raman et al., 2019). In 2018, the U.S. Food and Drug Administration (FDA) approved IDx-DR, the first AI-based device for detecting diabetic retinopathy (Yang et al., 2019). For glaucoma, DL algorithms measure the cup-to-disc ratio by analyzing the optic nerve head, while in macular degeneration, these technologies assess macular morphology to assist in diagnosis and grading (Dong et al., 2021). Such advancements not only improve diagnostic accuracy but also enhance therapeutic outcomes for patients.

Beyond diagnosis, AI systems are increasingly applied for disease prediction and progression forecasting. By analyzing longitudinal imaging data and clinical parameters, DL models can predict the risk of disease onset or progression before overt symptoms develop. For example, DL can estimate the likelihood of diabetic retinopathy worsening over time, enabling earlier intervention and personalized monitoring strategies (Dai et al., 2024). Similarly, predictive models for glaucoma can forecast future visual field loss based on baseline fundus images, aiding in timely treatment adjustments (Li et al., 2022). Such predictive capabilities empower ophthalmologists to adopt proactive management approaches, potentially preventing vision loss and improving long-term patient outcomes.

**FIGURE 1:**
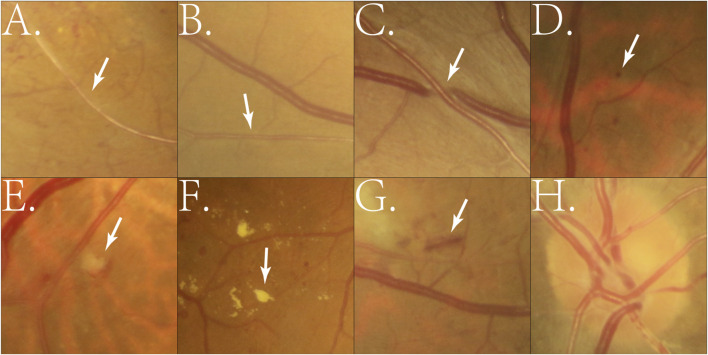
Example of manually cropped image patches containing hypertensive fundus lesions. **(A)** Arterial narrowing: Retinal arterioles become narrowed due to chronic hypertension-induced vascular wall thickening; **(B)** Reduced arteriovenous ratio: A decrease in the retinal artery-to-vein diameter ratio, reflecting arteriolar narrowing; **(C)** Arteriovenous nicking: Venous compression or deflection at arteriovenous crossings caused by thickened arteries; **(D)** Microaneurysm: Small round red dots (<125 μm) formed by capillary wall outpouchings due to microvascular damage; **(E)** Cotton wool spot: Small whitish patches representing localized retinal nerve fiber layer infarctions from ischemia; **(F)** Exudates: Yellowish deposits of lipids or proteins in the retina caused by vascular leakage; **(G)** Hemorrhage: Blood leakage into retinal layers, appearing as flame-shaped or blot-shaped lesions; **(H)** Optic disc edema: Swelling of the optic nerve head due to severe hypertension or increased intracranial pressure.

Hypertensive retinopathy is increasingly recognized as a marker for systemic vascular diseases, including cerebrovascular and cardiovascular conditions (Fraser-Bell et al., 2017; Rim et al., 2020; Wong et al., 2008). DL technologies have been employed to analyze CFP for tasks like retinal vessel segmentation, arteriovenous classification, and lesion detection. These algorithms precisely measure arteriovenous ratio (AVR), detect vascular narrowing, and identify microvascular abnormalities, providing critical insights for the early detection and grading of hypertensive retinopathy. Figure 1 illustrates representative examples of hypertensive fundus lesions, including arterial narrowing, AV nicking, and optic disc edema, etc.

Based on the above background, this review will summarize the research progress of DL in the analysis of CFP related to hypertension from the following aspects (Cheung et al., 2012): applications of DL in the analysis of hypertension-related retinal lesions (Wong and Mitchell, 2004), applications of DL in diagnosing hypertension-related ocular diseases, and (Wong and Mitchell, 2007) applications of DL in predicting hypertension-related diseases.

## 2 Retrieval methods

The search strategy included a combination of the following keywords: “deep learning,” “machine learning,” “artificial intelligence,” “convolutional neural network,” “fundus photography,” “retinal image,” “hypertension,” “hypertensive retinopathy,” “retinal vascular disease,” “retinal vein occlusion,” “arteriovenous ratio,” “retinal artery macroaneurysm,” “systemic disease prediction,” and “cardiovascular risk.”

The inclusion criteria were as follows: Original research articles published in peer-reviewed journals; Studies involving the application of DL or machine learning in fundus image analysis; Research specifically addressing the diagnosis, classification, or prediction of hypertension-related retinal or systemic diseases; Articles published in English between January 2010 and January 2025.

The exclusion criteria included: Studies not employing DL methods, such as those based solely on traditional statistical or machine learning techniques; Review articles, editorials, or case reports lacking original experimental data; Studies based on non-fundus imaging modalities (e.g., OCT, MRI, ultrasound) in which CFPs were not a central component.

**FIGURE 2:**
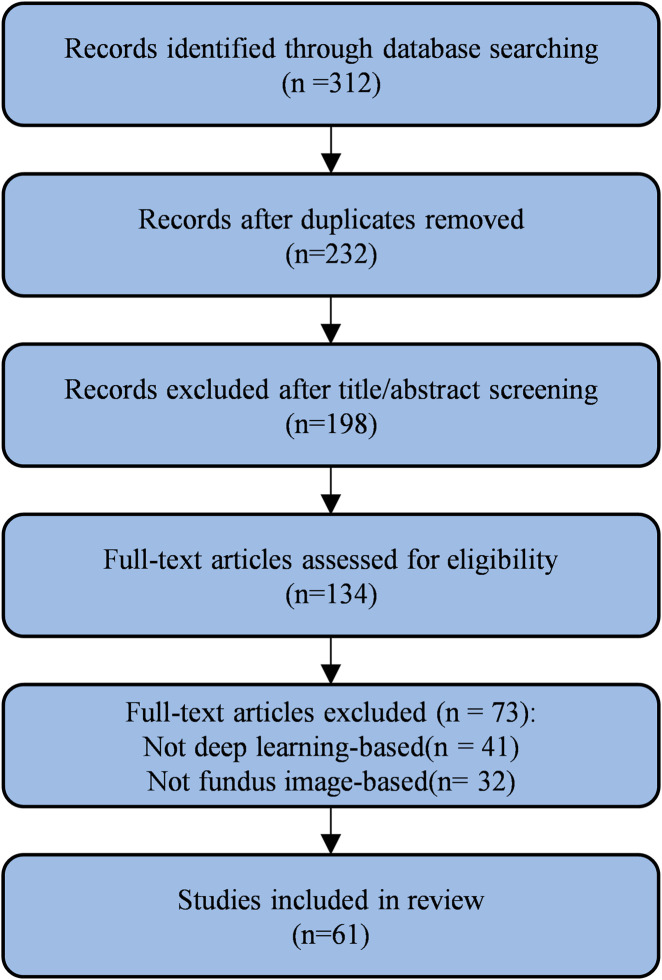
Document retrieval flow chart.

After removing duplicates, titles and abstracts were screened independently by two reviewers. Potentially relevant studies were retrieved for full-text evaluation. Discrepancies were resolved through consensus or consultation with a third reviewer. The screening and selection process is illustrated in Figure 2.

Based on the final selection, included studies were categorized according to the application scope of DL in hypertension-related fundus image analysis (Cheung et al., 2012): Application in analyzing hypertension-related retinal lesions (Wong and Mitchell, 2004), Application in diagnosing hypertension-related ocular diseases (Wong and Mitchell, 2007), Application in predicting hypertension and its systemic complications.

## 3 Applications of DL in the analysis of hypertension-related retinal lesions

Extensive studies on retinal microvascular phenotypes in CFPs have demonstrated that hypertension induces multiple abnormalities, including changes in vascular caliber and structure, as well as pathological features such as retinal hemorrhages, microaneurysms, and cotton wool spots (Dai et al., 2020).

### 3.1 Retinal vessel segmentation

Hypertension induces retinal vascular abnormalities, including pathological vessel growth and degeneration. Hypertensive retinopathy, a retinal disease closely linked to elevated and poorly controlled blood pressure, exacerbates vascular narrowing, particularly in small arteries. Retinal vessel segmentation, which refers to the pixel-wise delineation of blood vessels from fundus images, plays a pivotal role in diagnosing, treating, and planning surgeries for retinal diseases, serving as a fundamental step for precise visualization and quantification of vascular lesions (C et al., 2017). Vascular morphological changes, including shape, curvature, branching patterns, and width, are crucial markers for early disease detection (Abramoff et al., 2010). While manual vessel segmentation is accurate, it is labor-intensive, time-consuming, and heavily reliant on expert experience.

Deep learning-based retinal vessel segmentation in fundus imaging represents a cutting-edge technique that improves diagnostic precision and operational efficiency. By training deep neural network models, these methods autonomously learn vascular features and morphology, enabling precise segmentation of retinal vasculature. Commonly utilized datasets, such as STARE, DRIVE, CHASE_DB1, and HRF, facilitate the evaluation of these segmentation algorithms.

Early studies integrated DL with classical machine learning methods to enhance segmentation performance. For instance, Maji et al. (2015) proposed a hybrid architecture combining deep neural networks and random forests, achieving 93.27% accuracy and an area under curve (AUC) of 0.9195 on the DRIVE database. Despite these advancements, the generalization of their model to complex lesion regions required further improvement. Fu et al. (Fu et al., 2016; Xu et al., 2016) introduced convolutional neural networks (CNN) combined with fully connected conditional random fields (CRF) to refine segmentation results. Their DeepVessel system employed multi-scale feature integration, achieving notable performance improvements on DRIVE, STARE, and CHASE DB1 datasets (95.23%, 95.85%, and 94.89%, respectively). However, cross-dataset generalization and performance in extreme pathological regions remained challenges.

Further advancements in DL led to the development of fully convolutional neural networks (FCNN). Shelhamer et al. (2017) transformed fully connected layers into convolutional layers, enabling processing of inputs of varying sizes. Jiang et al. (2018) utilized FCNNs with transfer learning, simplifying the segmentation problem by focusing on regional vascular elements, thereby improving cross-database performance. Mo and Zhang, (2017) enhanced feature learning through deeply supervised FCNN, mitigating limited training data challenges via transfer learning. Oliveira et al. (2018) incorporated stationary wavelet transforms and multi-scale analysis to handle vessel width and directional variations, achieving superior results on standard datasets. However, high computational demands limited their direct application in resource-constrained environments.

To address boundary processing challenges, Zhou et al. (2017) introduced dense CRFs for enhancing vein contrast and smoothing boundary irregularities. Yan et al. (2018) improved segmentation accuracy for thin vessels by integrating pixel-level and segment-level losses. Residual structures and attention mechanisms also emerged as promising approaches. For instance, Li and Rahardja, (2021) proposed a residual U-Net optimized with activation-prior squeeze-and-excitation modules, while Zhao et al. (2021) developed a nested U-shaped network (NUA-Net) featuring multi-scale upsampling attention modules for robust segmentation of pathological images.

Efforts to enhance segmentation efficiency have shifted toward lightweight network designs. Chudzik et al. (2018) proposed a visual codebook framework combining CNNs and random forest embeddings, reducing computational complexity. Similarly, Raza et al. (2021) developed the DAVS-Net, which minimizes parameter requirements while maintaining high segmentation accuracy. Arsalan et al. (2021) designed shallow dual-stream networks (DSF-Net and DSA-Net) to enable efficient segmentation without traditional image enhancement. These lightweight models offer faster inference and lower computational cost, but may sacrifice fine-detail accuracy and generalizability in complex imaging scenarios.

To analyze the structural information of vascular networks more comprehensively, Yu et al. (2020) proposed a hierarchical partitioning framework based on graph algorithms. By classifying the hierarchical features of vascular bifurcations, this method offers a novel approach to quantifying lesion severity. However, the practical application of complex networks requires further investigation.

To address the segmentation of vessels at different scales, Tang et al. (2019) introduced the Multi-Proportion Channel Integration Model (MPC-EM), effectively solving segmentation problems for vessels of varying scales. Wang et al. (2020) designed the Hard Attention Network (HAnet), focusing on “hard-to-segment” areas. Wu et al. (2020) developed NFN+, which further refined vascular segmentation results through a multi-scale backbone network. These methods significantly improved the segmentation performance of small vessels and low-contrast regions but remain heavily reliant on large-scale datasets.

Table 1 summarizes DL-based methods for retinal vessel segmentation in hypertension-related analysis, including their architectures, datasets, and performance metrics. In summary, DL -based retinal vessel segmentation has significantly progressed through multi-scale feature learning, attention mechanisms, and lightweight designs. While existing methods achieve remarkable accuracy, challenges in extreme pathological regions, low-contrast images, and cross-dataset generalization persist. Future innovations will enhance these capabilities, providing robust support for early screening and precise diagnosis of retinal diseases.

**TABLE 1:** Deep learning-based retinal vessel segmentation methods for hypertension-related Analysis.

Authors	Architecture	Dataset	Accuracy	Sensitivity	Specificity	AUC
Maji at el. (2015)	random forest + DNN	DRIVE	0.933			0.920
Fu at el. (2016)	FCNN + CRF	DRIVE	0.947	0.729		
		STARE	0.955	0.714		
Xu et al. (2016)	CNN + CRF	DRIVE	0.952	0.760		
		STARE	0.959	0.741		
		CHASE DB1	0.949	0.713		
Jiang at el. (2018)	FCNN + TL	DRIVE	0.959	0.712	0.983	0.968
		STARE	0.965	0.782	0.980	0.987
		CHASE DB1	0.959	0.722	0.977	0.958
Mo and Zhang, (2017)	DSFCN	DRIVE	0.952	0.778	0.978	0.978
		STARE	0.967	0.815	0.984	0.989
		CHASE DB1	0.960	0.766	0.982	0.981
Oliveira et al. (2018)	SWT + FCNN	DRIVE	0.958	0.804	0.980	0.982
		STARE	0.969	0.832	0.986	0.991
		CHASE DB1	0.965	0.778	0.986	0.986
Zhou at el. (2017)	CNN + CRF	DRIVE	0.947	0.808	0.967	
		STARE	0.959	0.807	0.976	
		CHASE DB1	0.952	0.755	0.975	
		HRF	0.954	0.802	0.970	
Yan et al. (2018)	joint-loss DL framework	DRIVE	0.954	0.765	0.982	0.975
		STARE	0.961	0.758	0.985	0.980
		CHASE DB1	0.961	0.763	0.981	0.978
Li and Rahardja, (2021)	BSEResU-Net-33 + joint loss	DRIVE	0.957	0.832	0.976	0.982
		STARE	0.976	0.819	0.989	0.991
		HRF	0.964	0.807	0.980	
Chudzik et al. (2018)	CNN + VC	DRIVE		0.788	0.974	0.965
		STARE		0.827	0.980	0.984
Yu et al. (2020)	CNN	DRIVE	0.952	0.980	0.764	0.972
		STARE	0.961	0.982	0.784	0.979
Tang et al. (2019)	MPC-EM	DRIVE	0.957	0.856	0.971	0.982
		STARE	0.970	0.816	0.987	0.990
		CHASE DB1	0.965	0.811	0.981	0.985
		HRF	0.963	0.778	0.984	0.984
Wang et al. (2020)	HAnet	DRIVE	0.958	0.799	0.981	0.982
		STARE	0.967	0.819	0.984	0.988
		CHASE DB1	0.967	0.824	0.981	0.987
		HRF	0.965	0.780	0.984	0.984
Wu et al. (2020)	NFN+	DRIVE	0.958	0.800	0.981	0.983
		STARE	0.967	0.796	0.986	0.988
		CHASE DB1	0.969	0.800	0.988	0.989
Raza et al. (2021)	DAVS-NET	DRIVE	0.969	0.829	0.982	0.983
		STARE	0.974	0.824	0.987	0.988
		CHASE DB1	0.973	0.814	0.984	0.986
Arsalan et al. (2021)	DSF-NET	DRIVE	0.969	0.819	0.984	0.983
	DSA-NET	DRIVE	0.969	0.827	0.983	0.984
		STARE	0.970	0.861	0.980	0.987
		CHASE DB1	0.973	0.822	0.984	0.982

a. CHASE DB1: Child heart and health study in England database 1; b. CNN: convolutional neural network; c. CRF: conditional random field; d. DAVS: dense aggregation vessel segmentation; e. DL: deep learning; f. DNN: deep neural network; g. DRIVE: digital retinal images for vessel extraction; h. DSA: dual stream aggregation; i. DSF: dual stream fusion; j. DSFCN: deeply supervised fully convolutional network; k. EM: Expectation-maximization; l. FCNN: fully convolutional neural network; m. HRF: High-resolution fundus; n. MPC: Multi-scale parallel convolution; o. NET: network; p. NFN: network following network; q. STARE: structured analysis of the retina; r. SWT: stationary wavelet transform; s. TL: transfer learning; t. VC: vessel classification.

### 3.2 Retinal vessel classification and AVR calculation

Retinal vessel classification and AVR calculation are vital for evaluating the systemic impact of hypertension and related diseases. The AVR, a critical parameter representing the ratio of arterial to venous diameters in the retina, is closely linked to overall vascular health. Hypertension, for instance, can cause retinal arterial narrowing, resulting in a decreased AVR (Sun et al., 2009; Niemeijer et al., 2011) Classification, in this context, refers to the process of assigning each segmented vessel to either the arterial or venous category based on morphological and spatial features. Accurate AVR calculation depends on precise vessel segmentation and the correct classification of arteries and veins, which are fundamental steps in determining AVR (Miri et al., 2017). Small classification errors may cause significant deviations in AVR measurements, underscoring the importance of developing efficient and accurate classification methods.

Traditional approaches typically involve sequentially segmenting vessels before classifying arteries and veins. However, segmentation errors can propagate to the classification stage, reducing overall accuracy. To address these limitations, researchers have introduced multi-task learning frameworks that integrate segmentation and classification into a unified process, minimizing error propagation. Welikala et al. (2017) proposed a CNN-based model for the automatic classification of retinal arteries and veins. Unlike traditional methods reliant on handcrafted features, this approach directly learns patterns from data, enabling comprehensive and accurate classification across the entire retinal vascular system. Their method achieved an overall pixel-level classification accuracy of 86.97% and a segment-level accuracy of 85.24% on the UK Biobank dataset.

Xu et al. (2018) employed a fully convolutional neural network (FCNN) to simultaneously segment and classify arteries and veins, avoiding segmentation-induced error accumulation. On the DRIVE dataset, their method reached a sensitivity of 94.4%, specificity of 95.5%, and overall accuracy of 95.4%, demonstrating significant improvements compared to traditional sequential frameworks. Lepetit et al. (Lepetit-Aimon et al.) designed a large receptive field FCNN (LRFFCN), which expanded the network’s capacity to process complex vessel morphologies, outperforming traditional U-Net architectures in sensitivity and precision, particularly in challenging regions.

Traditional AVR calculation methods generally prioritize the widest vessels near the optic disc. However, studies suggest that hypertension predominantly affects smaller arteries, highlighting the limitations of localized vessel reliance in reflecting comprehensive disease impact (Niemeijer et al., 2011). To address this, Girard et al. (2019) developed a fully automated semantic segmentation method using a single encoder-decoder CNN to classify vessels as arteries or veins. This approach introduced a global AVR calculation method, leveraging data from the entire retinal field to improve accuracy and comprehensiveness.

Kang et al. (2020) designed AVNet, a novel segmentation network that enhances classification with a category-attention weighted fusion module and incorporates a vascular structure reconstruction algorithm, ensuring segmentation consistency. Their model achieved an accuracy of 94.9% for artery/vein classification. Similarly, Dai et al. (2020) employed vessel segmentation to exclude non-vascular features from retinal images, focusing solely on vascular structures. Using gradient-weighted class activation mapping (Grad-CAM), their lightweight CNN model identified hypertension-related microvascular abnormalities, particularly at branching points.

Morano et al. (2021) proposed an FCNN-based method for simultaneous segmentation and classification of arteries and veins. By decomposing the task into three sub-problems—artery segmentation, vein segmentation, and overall vessel segmentation—this approach produced comprehensive vessel maps and achieved superior performance, reaching a classification accuracy of 90.8% and a sensitivity of 91.2%.

Table 2 presents DL approaches for AVR estimation and structural analysis of retinal vasculature. Deep learning-based methods for retinal vessel segmentation and classification demonstrate substantial potential for enhancing AVR calculation accuracy. By incorporating multi-task learning, attention mechanisms, and graph-based optimizations, these methods effectively address the limitations of traditional approaches. Such advancements provide critical support for diagnosing and monitoring hypertension-related diseases, offering significant clinical utility.

**TABLE 2:** Deep learning approaches for arteriovenous ratio (AVR) estimation and retinal vascular structural analysis.

Authors	Architecture	Dataset	Accuracy	Sensitivity	Specificity
Welikala et al. (2017)	CNN	United Kingdom Biobank	0.870	0.861	0.877
Xu et al. (2018)	FCNN	DRIVE	0.900		
		INSPIRE-AVR	0.792		
Lepetit et al. (2018)	LRFFCN	DRIVE	0.810	0.778	0.844
Girard et al. (2019)	CNN 6D BP + LSP	DRIVE	0.865	0.863	0.866
		MESSIDOR	0.924	0.953	0.904
Dai et al. (2020)	CNN	segmented dataset	0.609		0.515
Kang et al. (2020)	AVNet + VSR	DRIVE	0.908	0.886	0.927
		LES-AV	0.922	0.943	0.909
		WIDE	0.938	0.925	0.951
Morano et al. (2021)	FCNN	RITE	0.892	0.875	0.909

a. AV: arteriovenous; b. AVR: arteriovenous ratio; c. BP: blood pressure; d. CNN: convolutional neural network; e. DRIVE: digital retinal images for vessel extraction; f. FCNN: fully convolutional neural network; g. LES: local enhancement strategy; h. LSP: local structure preservation; i. RITE: retinal image vessel tree extraction; j. United Kingdom: united kingdom; k. VSR: vessel structure reconstruction; l. WIDE: Wide-field fundus imaging; m. INSPIRE-AVR: INSPIRE, database for arteriovenous ratio; n. MESSIDOR: a database for retinal fundus imaging and diabetic retinopathy evaluation.

### 3.3 Retinal lesion segmentation and detection

The automated segmentation and detection of retinal lesions are essential for screening and diagnosing ophthalmic diseases. Detection, in this context, refers to the task of identifying and localizing specific pathological features—such as hemorrhages or microaneurysms—within fundus images, typically through bounding boxes or heatmaps. Retinal hemorrhages, microaneurysms, and cotton wool spots are key pathological features of hypertension and its complications, such as hypertensive retinopathy. Early detection and quantification of these lesions aid in disease intervention and treatment, providing critical diagnostic information for clinical research.

In single-lesion detection, researchers have proposed innovative solutions to address hemorrhages, microaneurysms, and exudates. For example, van Grinsven et al. (2016) introduced a selective data sampling strategy that optimized CNN performance in detecting hemorrhage regions. By training on misclassified negative samples, this method significantly reduced training time while improving detection precision. It achieved outstanding results in the KAGGLE and MESSIDOR datasets, with AUC values of 0.917 and 0.979, respectively. However, this method primarily targets single-lesion detection and lacks the capacity to extend to multi-lesion applications. Detecting microaneurysms is more challenging due to their small size and low contrast. Dai et al. (2018) developed a multi-sieving convolutional neural network (MS-CNN) guided by clinical reports. By integrating multimodal information, such as visual features and clinical reports, the model achieved high-quality detection and segmentation of microaneurysms. This model significantly improved segmentation accuracy across multiple datasets. For exudates, Prentašić and Lončarić, (2016) designed a ten-layer CNN architecture that enhanced detection capabilities in complex backgrounds by incorporating high-level knowledge of retinal structures, such as blood vessels and optic discs. However, the method relied heavily on manually engineered features, limiting its generalization to other lesion types.

To improve efficiency, researchers have developed multi-lesion detection and segmentation methods. Tan et al. (2017) proposed a single ten-layer CNN architecture to simultaneously segment exudates, hemorrhages, and microaneurysms, achieving automated multi-lesion detection and segmentation. Although the model demonstrated high pixel-level segmentation accuracy for three pathological features, its ability to detect smaller lesions (e.g., microaneurysms) requires improvement. Lam et al. (2018) combined sliding windows and cropped image patches, optimizing detection accuracy for hemorrhages and microaneurysms in small sample datasets. This approach achieved high pixel-level AUCs of 0.94 for hemorrhage detection and 0.95 for microaneurysm detection, effectively addressing resolution challenges in small sample learning. However, the computational cost of sliding windows limits its application in large-scale datasets.

To further enhance detection accuracy and robustness, researchers have incorporated data augmentation and multi-scale feature fusion strategies. Khojasteh et al. (2018) introduced a CNN embedded with preprocessing layers that enhanced image contrast and detail, improving detection performance for low-contrast lesions. Their model achieved a sensitivity of 0.990 and accuracy of 0.982 on the DIARETDB1 dataset. This method significantly improved classification accuracy for exudates, hemorrhages, and microaneurysms but relied heavily on preprocessing outcomes, requiring validation for noise robustness. Guo et al. (2019) developed a small-object segmentation network (L-Seg) to simultaneously segment soft exudates, hard exudates, microaneurysms, and hemorrhages. By integrating multi-scale feature fusion and multi-channel bucket loss (MCB Loss), L-Seg avoided misclassifications of small lesions as background or *vice versa*, achieving a mean AUC of 0.6515 on the IDRiD dataset and outperforming state-of-the-art models​. However, its complex network structure may limit practical clinical applications.

In recent years, researchers have gradually explored integrating lesion detection and disease grading tasks into a unified framework. Dai et al. (2021) developed a system named DeepDR for real-time image quality assessment, lesion detection, and DR grading. This system comprises three sub-networks: an image quality assessment sub-network, a lesion-aware sub-network, and a DR grading sub-network. The lesion-aware sub-network, trained on 10,280 labeled images, efficiently detects various lesions such as microaneurysms, cotton wool spots, hard exudates, and hemorrhages, achieving AUCs of 0.901, 0.941, 0.954, and 0.967, respectively. This integrated framework is not only applicable to diabetic retinopathy but also provides valuable references for pathological feature extraction and grading in hypertensive retinopathy.

Recent studies have extended the application of DL to detect various hypertensive-induced retinal lesions. Ghenciu et al. (2024) proposed a multi-task DL framework capable of simultaneous vessel segmentation, lesion detection, and disease risk assessment. Their model effectively identified pathologic features closely associated with hypertension, such as arterial narrowing and microvascular abnormalities, and further estimated systemic risk based on these image-derived features.

Table 3 details DL models developed for detecting retinal lesions in hypertension-related diseases, including microaneurysms, hemorrhages, and exudates. Deep learning-based methods for retinal lesion detection and segmentation have demonstrated significant potential in diagnosing hypertensive retinopathy and related systemic diseases. From precise detection of single lesions (e.g., microaneurysms, exudates, and hemorrhages) to the joint segmentation of multiple lesions and the integration of detection and grading frameworks, these approaches have substantially enhanced the automation and performance of retinal lesion detection, offering effective tools for the early diagnosis and monitoring of hypertension and its complications.

**TABLE 3:** Deep learning models for lesion detection in hypertension--related retinal diseases.

Authors	Architecture	Dataset	Lesion	Accuracy	Sensitivity	Specificity	AUC
van Grinsven et al. (2016)	CNN	KAGGLE	Microaneurysms		0.837	0.851	0.894
		MESSIDOR	Microaneurysms		0.919	0.914	0.972
Dai et al. (2018)	MS-CNN	DIARETDB1	Haemorrhages	0.961			
Prentasic and Loncaric, (2016)	CNN	DRiDB	Exudates		0.780		
Tan et al. (2017)	CNN	CLEOPATRA	Microaneurysms		0.461	0.980	
			Haemorrhages		0.626	0.989	
			Exudates		0.876	0.987	
Lam et al. (2018)	CNN	e-Ophta	Microaneurysms				0.940
			Exudates				0.950
Khojasteh et al. (2018)	CNN	DIARETDB1	Microaneurysms	0.788	0.840	0.970	
			Haemorrhages	0.825	0.800	0.940	
			Exudates	0.910	0.910	0.940	
Guo et al. (2019)	L-Seg	DRiDB	Microaneurysms				0.463
			Haemorrhages				0.673
			Hard Exudates				0.795
			Soft Exudates				0.711
		DDR	Microaneurysms				0.105
			Haemorrhages				0.359
			Hard Exudates				0.555
			Soft Exudates				0.265
Dai et al. (2021)	DeepDR	SIM cohort	Microaneurysms		0.880	0.733	0.901
			Haemorrhages		0.932	0.880	0.967
			Hard Exudates		0.905	0.858	0.954
			Soft Exudates		0.900	0.831	0.941

a. CNN: convolutional neural network; b. MS: Multi-Scale; c. L-Seg: an end-to-end model for multi-lesion segmentation in fundus imaging; d. KAGGLE: Kaggle EyePACS, diabetic retinopathy detection dataset; e. MESSIDOR: a database for retinal fundus imaging and diabetic retinopathy evaluation; f. DIARETDB1: Diabetic retinopathy database 1; h. DRiDB: diabetic retinopathy image database; i. CLEOPATRA: CLEOPATRA, retinal image database; j. e-Ophta: a publicly available set of retinal fundus images developed for diabetic retinopathy lesion detection. k. DDR: diabetic retinopathy detection database; l. SIM, cohort: Singapore integrated medical cohort.

## 4 Applications of DL in diagnosing hypertension-related ocular diseases

Hypertension exerts significant effects on the fundus vasculature, characterized by vascular wall thickening and hardening, which reduce elasticity and restrict blood flow. These vascular changes further exacerbate hypoxia and nutrient deprivation in ocular tissues. Additionally, hypertension-induced inflammation damages vascular endothelial cells, increasing vascular permeability and causing leakage. These pathological changes impair the retina’s blood supply, oxygenation, and waste clearance functions, contributing to fundus diseases such as retinal vein occlusion (RVO) and ischemic optic neuropathy (Fraser-Bell et al., 2017).

In recent years, numerous DL-based automated diagnostic systems have been developed to analyze CFP and identify various lesions. These systems, trained on extensive labeled datasets, autonomously learn lesion features, enabling efficient and accurate disease diagnosis while offering robust clinical support.

### 4.1 Retinal vein occlusion (RVO)

Hypertension is a primary risk factor for RVO, one of the most prevalent retinal vascular diseases threatening vision. Fundus manifestations of RVO include superficial retinal hemorrhages, vascular dilation and tortuosity, cotton wool spots, and optic nerve edema caused by elevated venous pressure (Song et al., 2019). Traditional RVO diagnosis relies on expert interpretation, but DL offers a more efficient and objective approach.

Ren et al. (2022) developed a DL-based artificial intelligence algorithm to differentiate RVO patients from healthy individuals by analyzing fundus photographs. The CNN achieved sensitivity of 0.8367, specificity of 0.9810, and accuracy of 0.9534, showcasing exceptional diagnostic capabilities as an auxiliary screening tool. Xu et al. (2022) further proposed an intelligent diagnostic system capable of determining RVO presence and classifying the disease based on the occlusion site. By incorporating ResNet18 and the Coordinate Attention (CA) module, this system’s diagnostic performance closely aligned with clinical evaluations, highlighting the potential of DL in RVO subtype diagnosis.

According to a study published in 2024, Masayoshi et al. (2024) developed a CNN-based classification model trained on multi-center datasets for detecting and grading RVO severity. The model achieved high accuracy in distinguishing branch and central RVO and was shown to be effective in real-world clinical settings. Its robustness across diverse datasets suggests strong generalizability, making it a viable tool for aiding ophthalmologists in large-scale screening of hypertensive retinal complications.

In addition, a more recent study by Wan et al. (2023) introduced the Swin Transformer for RVO diagnosis, which demonstrated excellent results in classifying different types of RVO, including macular retinal vein occlusion (MRVO), central retinal vein occlusion (CRVO), and branch retinal vein occlusion (BRVO). The Swin Transformer model achieved an overall accuracy of 98.75% and outperformed other models in all tested categories, including MRVO, CRVO, BRVO, and normal classification. Notably, the model’s performance in detecting MRVO was exceptional, with a sensitivity of 93.89% and specificity of 99.98%. Using label smoothing to reduce overfitting, the Swin Transformer further enhanced its generalization ability, demonstrating its potential as a reliable tool for automated RVO diagnosis. This approach marks a significant advancement in DL-based RVO detection, offering the potential for more accurate and efficient diagnoses in clinical settings.

### 4.2 Ischemic optic neuropathy (ION)

ION, including arteritic anterior ischemic optic neuropathy (AAION) and non-arteritic anterior ischemic optic neuropathy (NAION), significantly affects visual function and is closely associated with hypertension. Rapid and accurate differentiation between AAION and NAION is clinically critical, as treatment approaches vary considerably (Biousse and Newman, 2015).

Recent advancements in DL have enabled effective automated diagnostic systems for ION based on fundus photography. Liu et al. (2023) developed a deep learning system named ONION using the EfficientNet-B0 architecture. This model leveraged compound scaling to balance network depth, width, and resolution, making it computationally efficient and suitable for deployment in real-world settings. ONION successfully distinguished acute optic neuritis from NAION, achieving an area under the curve (AUC) of 0.902, sensitivity of 0.814, and specificity of 0.841, which were comparable to expert ophthalmologists’ evaluations. However, the model’s reliance on a single fundus view may limit contextual interpretation in complex cases.

Further advancements by Gungor et al. (2024) utilized a DL system DLS trained on a multi-center dataset using a customized CNN architecture to differentiate AAION from NAION. Their approach integrated class activation mapping (CAM) for visual explain ability and localized lesion interpretation. The model attained high diagnostic accuracy (0.926), sensitivity (0.911), and specificity (0.934), demonstrating strong generalizability across diverse imaging sources. The key advantage of this method lies in its clinical interpretability and robust performance; however, the need for high-quality annotated data may limit scalability in under-resourced settings.

Additionally, Liu et al. (2021) implemented a ResNet-152-based DL model to detect a variety of optic disc abnormalities, including ischemic optic neuropathy. The deep residual network structure facilitated gradient flow and enabled learning of complex hierarchical features. This approach yielded high diagnostic accuracy, with an overall AUC of 0.87, sensitivity of 0.90, and specificity of 0.69 on external datasets. While the model showed strong sensitivity, its relatively lower specificity suggests susceptibility to false positives and indicates potential for further refinement.

Another recent study applied a modified DenseNet architecture for multi-disease classification involving ION (Pachade et al., 2025). This model utilized dense connections to improve feature reuse and mitigate vanishing gradients in deep layers, enhancing performance on small and imbalanced datasets. Although the approach demonstrated favorable results in internal validation, limited external validation restricts conclusions about generalizability.

These DL-driven diagnostic advancements in ION underline the potential of automated systems to improve diagnostic accuracy and clinical decision-making, especially in differentiating between arteritic and non-arteritic forms of the disease, thereby facilitating timely and appropriate clinical interventions.

In addition to RVO and ION, age-related macular degeneration (AMD) has also been reported to share risk factors with hypertension (Age-Related Eye Disease Study Research G, 2000; Miyazaki et al., 2003; Fraser-Bell et al., 2005). Although AMD is primarily a degenerative disease affecting the macula rather than a vascular disorder, some studies suggest that hypertension-induced changes in choroidal circulation may exacerbate AMD development. Given the differences in disease mechanism and manifestation compared to hypertensive retinal vasculopathies, AMD was not a major focus of this review. Nevertheless, recent DL models such as HCSP-Net and SCSP-Net have demonstrated high accuracy in AMD classification tasks, highlighting the broader potential of DL in fundus image-based diagnosis beyond vascular pathologies (Wan et al., 2024).

## 5 Applications of DL in predicting hypertension-related diseases

Retinal blood vessel structural changes are critical early indicators of hypertension and cardiovascular diseases, with retinal arteriolar narrowing extensively studied as a precursor to hypertension (Wang et al., 2008; Kawasaki et al., 2009). Research indicates that individuals with normal blood pressure but narrowed retinal arterioles are more likely to develop hypertension, and patients with mild hypertension are prone to progress to severe hypertension (Smith et al., 2004). CFPs features reflecting vascular health—such as vessel caliber, branching patterns, tortuosity, AVR, and microvascular abnormalities—are closely associated with hypertension onset and progression. Deep learning models that automate feature extraction and classifier construction provide novel technological means for diagnosing and predicting hypertension.

Zhang et al. (2020) utilized the Inception-v3 neural network to analyze CFPs and successfully developed a DL model for hypertension prediction. The model achieved a diagnostic accuracy of 68.8% and an AUC of 0.766, demonstrating the potential to predict hypertension solely using CFPs. However, further improvements in specificity and sensitivity are required, and future research should focus on optimizing algorithms and model architectures.

Beyond hypertension, CFPs also provide insights into cardiovascular health and future disease risks. Retinal microvascular features are closely associated with cardiovascular disease risk, offering critical information for systemic disease prediction. Poplin et al. (2018) trained a model to predict major adverse cardiovascular events (MACE) within 5 years using CFPs. The model achieved an AUC of 0.70, which improved to 0.72 when integrated with the European SCORE risk calculator, underscoring the complementarity of DL models and traditional risk assessment tools.

Further advancements have been made in personalized cardiovascular health evaluations using DL. Gerrits et al. (2020) developed a model to predict cardiometabolic risk factors from CFPs, achieving robust performance across various age and gender groups, thus enabling personalized cardiovascular health assessments. Ma et al. (2022) designed a DL algorithm to predict the 10-year risk of ischemic cardiovascular disease (ICVD) in the Chinese population using CFPs. Their model achieved exceptional validation results, with AUCs of 0.971 and 0.976 in internal validation and 0.859 and 0.876 in external validation, confirming its applicability across diverse populations. Additionally, Diaz-Pinto et al. (2022) proposed a method combining multi-channel variational autoencoders (mcVAE) and a deep regression model to estimate left ventricular mass and end-diastolic volume. Their approach successfully predicted myocardial infarction risk, achieving an AUC of 0.80, a sensitivity of 0.74, and a specificity of 0.71, highlighting the potential of fundus imaging in assessing cardiac structural abnormalities.

Zhou et al. (2024) designed a multimodal DL model integrating CFPs and clinical features (e.g., blood pressure, BMI) to predict preeclampsia. Remarkably, CFP-based prediction alone achieved considerable accuracy, and performance was further enhanced with multimodal data fusion. This approach highlights the feasibility of using retinal biomarkers as non-invasive tools for early identification of high-risk pregnant individuals, reinforcing the role of fundus AI systems in obstetric care.

CFPs offers a non-invasive, cost-effective solution for predicting hypertension and cardiovascular diseases. DL-based models accurately extract and quantify retinal microvascular changes, enabling early disease detection and risk assessment. Nonetheless, current models require optimization for cross-population generalizability, low-quality image processing, and enhanced prediction comprehensiveness.

## 6 Discussion

Hypertension, as a systemic disease, induces extensive pathological changes in retinal vasculature, including arteriovenous narrowing, vascular wall hardening, microaneurysm formation, and vascular leakage (Fraser-Bell et al., 2017). These changes not only affect the local blood supply and oxygenation of the retina but are also closely associated with hypertensive retinopathy, retinal vein occlusion, and age-related macular degeneration. Therefore, early detection and risk prediction of hypertension-induced retinal lesions hold significant clinical value.

DL-based CFP analysis has developed rapidly in recent years. Leveraging the learning capabilities of neural networks, these technologies identify and extract various information from CFPs using large training datasets. DL demonstrates exceptional potential in retinal vessel segmentation, lesion detection, disease classification, and prediction. By utilizing high-quality annotated CFP data and advanced DL algorithms such as CNN, U-Net, and ResNet, these techniques efficiently learn and extract structural features and pathological patterns associated with diseases, providing reliable support for diagnosing and predicting hypertension and related conditions.

Retinal vessel segmentation and classification are foundational for assessing the impact of hypertension on microvasculature. Studies have shown that retinal arteriolar narrowing is closely related to hypertension progression, and a decrease in the AVR serves as an early indicator of hypertension and related cardiovascular diseases. Traditional methods of vessel segmentation and classification rely on expert manual annotation, which is time-consuming and prone to subjective bias. Recent advances in DL have significantly improved segmentation and classification accuracy through multi-task learning and feature fusion. For example, the graph algorithm framework proposed by Yu et al. (2020) enables hierarchical quantification of vascular bifurcation features, while Kang et al.'s AVNet integrates attention-weighted fusion modules to enhance arteriovenous classification precision (Kang et al., 2020). Additionally, models combining FCNN and multi-scale feature extraction have demonstrated superior performance in detecting small vessels and processing low-contrast images (Morano et al., 2021).

Hypertension-induced retinal lesions, such as microaneurysms, hemorrhages, cotton wool spots, and exudates, are critical indicators for clinical diagnosis. DL models can automatically detect and classify these lesion features. Guo et al.'s L-Seg model achieved precise segmentation of soft exudates, hard exudates, microaneurysms, and hemorrhages through multi-scale feature fusion and loss optimization (Guo et al., 2019). Similarly, Dai et al.'s DeepDR system integrates lesion detection and disease grading, providing comprehensive assessments of hypertension-related retinal lesions (Dai et al., 2021). Despite achieving high detection accuracy, current models still require optimization in generalizability for small datasets and robustness in complex lesion areas.

Early screening of hypertensive retinopathy is crucial for preventing vision loss and assessing systemic cardiovascular disease risks. By analyzing morphological changes in retinal vasculature (e.g., vessel caliber, tortuosity, bifurcation angles), DL models can achieve automated hypertension diagnosis and risk prediction. Recent studies have applied DL-based vessel segmentation to quantify retinal vascular morphology in hypertension screening. Shi et al. (2024) developed an automated analysis system using CFP and DL to measure features such as branching angle, vessel diameter, fractal dimension, and tortuosity. Their findings revealed that hypertension was associated with increased tortuosity, while cardiovascular disease correlated with larger vessel diameters. These vascular features may serve as non-invasive indicators of systemic vascular risk in hypertensive patients. For instance, Poplin et al. (2018) developed a DL model that predicts major adverse cardiovascular events (MACE) using CFPs, achieving accuracy comparable to traditional risk assessment tools. Additionally, Ma et al.'s algorithm demonstrated excellent performance in predicting 10-year ischemic cardiovascular disease risk in the Chinese population, providing valuable support for personalized health management (Ma et al., 2022).

Advancements in DL–based automated analysis of CFPs have significantly alleviated the workload of ophthalmic professionals. These models enable rapid and accurate segmentation of retinal vasculature and identification of pathological lesions, facilitating efficient and comprehensive assessment of hypertension-related ocular and systemic manifestations. In resource-constrained settings with limited access to ophthalmology specialists, such technologies offer scalable and reliable solutions for screening and diagnosis. Automated algorithms can analyze CFPs to detect early vascular changes, allowing for timely intervention and reduction of hypertensive complications. A recent community-based study conducted in China further demonstrated the value of AI-assisted systems in screening and referral for DR. Using a DL model trained on CFPs, the system achieved high sensitivity (93.6%) and specificity (78.5%) in detecting DR, and outperformed community physicians in identifying cases requiring referral (Dong et al., 2022). Notably, the AI system provided diagnostic results and referral recommendations within seconds, significantly improving screening efficiency and reducing the burden on non-specialist clinicians​. These findings support the feasibility of deploying similar AI frameworks for hypertension-related ocular screening in resource-limited settings. Consequently, these tools empower primary healthcare facilities to deliver more accessible and effective hypertension management.

Telemedicine also stands to benefit substantially from these developments. Patients can capture and upload CFPs through digital platforms, enabling remote clinicians to perform automated image analysis. This reduces both the time and cost of in-person consultations. Furthermore, remote evaluation facilitates early detection and personalized treatment recommendations, supporting effective hypertension control in home-based care. Nevertheless, the successful implementation of such applications depends on addressing challenges related to technical infrastructure, algorithmic robustness, and data privacy. Ensuring system security, standardizing data management, and establishing clear privacy protocols remain critical.

In addition to CFP-based models, deep learning systems have also demonstrated strong potential in other ophthalmic imaging modalities, offering valuable insights for future diagnostic applications. For example, Xu et al. (2020) developed a weakly supervised model for detecting central serous chorioretinopathy (CSCR) from OCT B-scan images by combining local binary patterns with discrete wavelet transform, achieving high diagnostic accuracy without the need for detailed pixel-level annotations. Similarly, an EfficientNet-B6-based system was designed to classify pterygium severity from anterior segment photographs, reaching 94.68% accuracy and showcasing the feasibility of lightweight AI models in resource-constrained environments (Xu et al., 2021). Building upon such advancements, OCT and OCT angiography (OCTA) have emerged as valuable complementary tools to CFP, particularly for assessing retinal structural changes in systemic diseases. DL applications using OCT have enabled automated detection of macular edema, subretinal fluid, and postoperative changes, with several studies from China confirming the effectiveness of CNNs in anterior segment analysis (Duan and Zhang, 2022). Although direct evidence linking OCT-based AI models to hypertension-related retinal changes remains limited, these findings collectively underscore the versatility of DL across imaging modalities and its growing role in comprehensive ocular diagnostics.

Moreover, the field of intelligent ophthalmology (IO) in China has seen significant growth, driven by the increasing integration of AI technologies in ophthalmic care. According to recent research, the development of AI-driven diagnostic tools in China has seen major technological breakthroughs, especially in the analysis of retinal diseases such as diabetic retinopathy, glaucoma, and age-related macular degeneration. The collaboration between medical institutions and AI experts has accelerated the creation of diagnostic platforms that now outperform traditional diagnostic methods in terms of speed, precision, and scalability (Gong et al., 2024).

Despite the promising advancements, several challenges continue to hinder the widespread clinical adoption of AI-based CFP analysis. Variability in data quality and cohort diversity limits the generalizability of models across different populations. Data distribution bias—such as differences in image resolution, disease prevalence, and patient demographics—can significantly degrade model performance when tested on external datasets. Cross-dataset evaluations have demonstrated AUC drops of up to 10%–15% when models trained on single-center data are applied to independent cohorts, emphasizing the need for more robust training strategies. Annotation inconsistency, stemming from subjective labeling or varying clinical standards, further contributes to performance variability and limits reproducibility.

The computational complexity of deep learning algorithms, along with their substantial resource requirements, poses significant barriers to real-time deployment—particularly in low-resource or remote healthcare environments. Additionally, ensuring patient data privacy, algorithmic transparency, and compliance with regulatory frameworks remains a pressing concern.

Moreover, current deep learning models exhibit several notable limitations, including overfitting on small or imbalanced datasets, limited interpretability in clinical decision-making, and susceptibility to domain shifts when applied to images from different devices or populations. Although some multi-center studies, such as those by Masayoshi et al. (2024) and Gungor et al. (2024), have demonstrated improved generalizability by incorporating diverse datasets, many models still lack rigorous external validation. Clinical application cases, including the FDA-approved IDx-DR system for diabetic retinopathy screening (Yang et al., 2019), have shown that AI deployment can increase diagnostic sensitivity, suggesting that similar integration efforts are needed for hypertension-related fundus diseases. Addressing these limitations is critical for the safe, effective, and sustainable translation of DL-based fundus image analysis into clinical workflows.

Equally important are the ethical and societal considerations surrounding the deployment of AI in ophthalmology. Protecting patient privacy, mitigating algorithmic bias across demographic groups, and ensuring fairness in model performance are essential prerequisites for clinical acceptance and trust. As AI systems increasingly inform diagnostic decisions, future research must prioritize explainability, ethical governance, and the development of diverse, representative datasets to support responsible and equitable AI integration into clinical practice.

Future directions should prioritize (Cheung et al., 2012): development of unified frameworks with cross-dataset adaptability to enhance generalization (Wong and Mitchell, 2004); integration of multimodal imaging data—such as OCT and OCTA—to improve diagnostic performance and risk stratification (Wong and Mitchell, 2007); optimization of lightweight architectures for deployment in low-resource and telemedicine settings (Bruce et al., 2011); establishment of comprehensive computer-aided diagnosis (CAD) systems combining segmentation, detection, classification, and prediction into a single, efficient platform (Rodenbeck and Mackay, 2019); exploration of privacy-preserving learning methods, such as federated learning, to enable decentralized model training while safeguarding patient data; and (Romero-Oraa et al., 2020) development of real-time and portable AI solutions using edge computing technologies, such as deploying lightweight models on devices like NVIDIA Jetson, to facilitate on-site fundus analysis and broaden access to diagnostic services in primary care and remote environments.

## 7 Conclusion

The diagnostic and predictive technologies for hypertension-related diseases based on CFPs and DL are gradually transforming traditional medical practices. By continuously optimizing model structures, expanding datasets, and exploring multimodal integration methods, these technologies hold the promise of achieving more accurate personalized diagnosis and health management. They provide more efficient and convenient medical services for hypertension patients while alleviating the burden on primary healthcare facilities and physicians. With the proliferation of DL technologies and fundus imaging devices, these advancements are expected to play an increasingly important role in clinical diagnosis, telemedicine, and health management. They offer new solutions for disease prevention and treatment, paving the way for improved patient outcomes and more efficient healthcare delivery systems.

## Abbreviations

HR, hypertensive retinopathy; OCT, optical coherence tomography; OCTA, optical coherence tomography angiography; FFA, fundus fluorescein angiography; AI, artificial intelligence; DL, deep learning; AVR, arteriovenous ratios; CNN, convolutional neural network; CRF, conditional random field; NUA-Net, nested U-shaped network; RVO, retinal vein occlusion; MRVO, macular retinal vein occlusion; CRVO, central retinal vein occlusion; MACE, major adverse cardiovascular events; mcVAE, multi-channel variational autoencoders; IO, intelligent ophthalmology; CAD, computer-aided diagnosis; NM, normal macula; DMD, dry macular degeneration; WMD, wet macular degeneration.
